# Advances in Rheology and Polymer Processing

**DOI:** 10.3390/polym18172130

**Published:** 2026-09-01

**Authors:** Célio Fernandes, Luís Lima Ferrás, Alexandre Miguel Afonso

**Affiliations:** Center for Studies of Transport Phenomena (CEFT), Department of Mechanical Engineering, Faculty of Engineering, University of Porto, 4200-465 Porto, Portugal; lferras@fe.up.pt (L.L.F.); aafonso@fe.up.pt (A.M.A.)

## 1. Editorial: Rheology and Polymer Processing

The rheology of polymer materials is the decisive link between molecular/mesoscale structure and macroscopic manufacturing outcomes. In industrial operations such as extrusion, injection and blow molding, film casting, and fiber spinning, rheological properties govern pressure drop, mixing and dispersion, interfacial stability, flow instabilities, and flow-induced orientation and crystallization. Understanding and manipulating rheological behavior is therefore essential for defining robust processing windows, increasing product quality, and enabling efficient manufacturing [1,2,3,4,5,6,7,8,9,10,11].

This Special Issue on rheology and polymer processing highlights recent advancements spanning novel experimental techniques, theoretical and phenomenological models, and computational simulations, which enhance our understanding of polymer flow behavior and its impact on processing. The contributions collectively emphasize an increasingly tight loop among rheological characterization under processing-relevant conditions, predictive modeling, and validation using realistic process geometries [12,13,14,15,16].

## 2. Recent Developments of Rheology–Processing Interface

### 2.1. From Linear Viscoelasticity to Nonlinear, Process-Relevant Rheology

Polymer rheology is shifting from small-amplitude linear viscoelasticity toward nonlinear rheological measures that more accurately represent manufacturing flows. Large-amplitude oscillatory shear, transient protocols (e.g., start-up, stress growth, and relaxation), and combined-mode testing are increasingly being used to examine strain hardening/softening, yielding, thixotropy, and other nonlinear signatures that correlate with processability and defect formation [5,17,18,19].

### 2.2. Complex Formulations: Multiphase and Filled Polymer Systems

Many industrial materials are blends or composites containing fillers, fibers, and functional additives. Their rheology reflects coupled relaxation processes (matrix, interphases, and filler networks) and strong sensitivity to thermal/mechanical history. Recent studies have improved our ability to connect dispersion state and interfacial interactions to measurable rheological fingerprints and to practical aspects such as flow-induced phase morphology, die swell, and melt fracture [9,14,20,21,22,23,24].

### 2.3. Computational Rheology and Multiscale Modeling

Computational tools continue to expand in scope and accessibility. Multiscale approaches that link chain-level dynamics to continuum constitutive forms, as well as finite-element/finite-volume simulations informed by rheological measurements, are increasingly being used for virtual prototyping and process optimization [6,12,25,26,27]. In parallel, reduced-order, surrogate, and data-driven models are being explored to accelerate parameter identification and sensitivity analyses, provided that physical constraints and uncertainty are treated carefully [15,16,28].

## 3. Remaining Knowledge Gaps

Despite progress, persistent challenges motivate continued research [24,29,30,31].

- Nonlinear rheology under coupled thermal and pressure histories: Processing flows couple deformation, temperature, and pressure. Quantitative datasets and models that remain predictive across these coupled conditions are still limited.
- Extensional and mixed-flow measurements for complex systems: many instabilities and defects are driven by extensional or mixed deformations; yet, routine, intercomparable measurements are scarce, particularly for multiphase and highly filled materials.
- Numerical schemes that robustly resolve viscoelastic flow instabilities: capturing elastic instabilities, melt fracture, and transient free-surface phenomena in realistic processing geometries requires more stable high-Weissenberg-number algorithms, adaptive resolution, and consistent stabilization that does not ignore the physics [32,33].
- Constitutive models informed by molecular/particle simulations: direct molecular simulations (e.g., coarse-grained MD, Brownian dynamics, and slip-link/single-chain models) are increasingly needed to guide the development of mesoscopic constitutive models that can be coupled to continuum solvers and validated against nonlinear flow protocols [25,34].
- Thermodynamically consistent rheological modeling: embedding constitutive equations within nonequilibrium thermodynamics frameworks helps enforce dissipation and avoids unphysical behavior (e.g., negative viscosities, spurious energy generation, and loss of realizability) [34,35,36].
- Multiscale modeling across the hierarchy: a continuing need is for workflows that link polymer structure and composition (chemistry, architecture, and polydispersity) to mesoscopic dynamics and, ultimately, to macroscopic rheology and process outcomes in a process that is quantitatively traceable [12,25].
- Morphology-aware rheology in complex soft materials: processing often occurs in materials with evolving, multcomponent morphologies (e.g., hydrogels, semicrystalline polymers and flow-induced crystallization, soft–hard nanocomposites, and worm-like micellar/surfactant solutions), where rheology is strongly coupled to structure formation, breakage, and anisotropy [37,38,39,40,41].
- Bridging rheology to product quality metrics: translating material functions into actionable predictions for surface finish, dimensional stability, anisotropy, weld-line integrity, and long-term performance remains an open problem.
- Validation and uncertainty quantification: both mechanistic and data-driven models require systematic validation and uncertainty quantification to be dependable in industrial design loops.

## 4. Contributions to This Special Issue

This Special Issue collects eight papers. Rather than reiterating each contribution in detail, we highlight a few representative themes that connect this Special Issue to broader developments in polymer rheology and processing.

- Process-intensification and realistic flow physics in extrusion and molding: process-scale modeling is advancing toward more realistic forcing and boundary conditions, including vibration-assisted twin-screw extrusion and ultrasonic micro-injection molding analyzed via dimensionless groups [11,12,42,43].
- Rheology of complex formulations and industrial compounds: several contributions focus on how additives and formulation chemistry reshape viscoelasticity and processability, spanning cationic polyacrylamide solutions, pigment masterbatches, and modified polyamides [44,45,46].
- Rheology-informed design for additive manufacturing of polymers and composites: material-extrusion printing continues to benefit from rheology that connects shear/extension, interlayer bonding, crystallization, and filler effects to printability and properties, including silica-filled PLA filaments and functionally graded PEEK components [47,48,49,50].
- Processing–structure relationships across length scales: overview and perspective studies emphasize how processing histories create structure (and thus performance), reinforcing the need to connect molecular/mesoscale models with processing-relevant measurements [1,17,25,51].

Together, the contributions in this Special Issue reflect the field’s dual trajectory: increasingly sophisticated characterization and simulation of nonlinear flow and a growing need to translate rheological descriptors into robust, process-specific quality metrics.

## 5. Perspectives and Future Research Directions

Several research directions appear especially relevant.

- Rheology of sustainable and circular polymers: Biobased polymers and mechanically/chemically recycled streams broaden the distributions in molecular architecture, additives, and contaminants. Rheology can support rapid, process-relevant fingerprints for quality control and for linking reprocessing histories to performance [52,53,54,55,56,57].
- Advanced processing technologies: Microfluidics, electrospinning, and additive manufacturing require extreme confinement, high deformation rates, and strong field coupling. New rheometric concepts and boundary condition measurements (e.g., slip and wall adsorption/depletion layers) are needed to build predictive models [29,30,49,58].
- Instability-resolving computational rheology: progress in predictive process simulation will depend on numerically robust formulations and discretizations that can access high elasticities while preserving the onset and nonlinear development of elastic instabilities [32,33].
- Thermodynamics-guided constitutive modeling: Future constitutive development should increasingly incorporate nonequilibrium thermodynamics structure (e.g., dissipation inequalities and consistent driving forces) to maintain stability and physical realizability when embedded in flow solvers [34,35,36].
- Molecular-to-continuum multiscale workflows: A key opportunity is the systematic coupling among molecular/particle simulations, mesoscopic constitutive modeling, and continuum process simulation, enabling model forms and parameters to be constrained by microphysics rather than fitted ad hoc [25,34].
- Morphology-aware processing models for structured materials: for many emerging and industrially important systems (hydrogels, semicrystalline polymers, nanocomposites, and worm-like micellar solutions), predictive processing requires models that track morphology evolution and its feedback on rheology [37,38,39,40,41].
- Integrated rheology with in situ structure characterization: combining rheometry with scattering, imaging, spectroscopy, and thermal analysis under flow will further clarify how deformation histories control morphology evolution (e.g., crystallization, phase separation, and filler-network development) [6,13,59,60].
- Digital-twin workflows for polymer processing: coupling computational rheology with uncertainty quantification and surrogate modeling can enable faster optimization and, eventually, real-time process control using in-line sensor feedback [15,61,62,63].

## 6. Conclusions

The rheology of polymeric materials remains central to polymer processing science and engineering. By focusing on process-relevant characterization, predictive modeling, and computational simulation, this Special Issue aims to further our understanding of rheology–processing relationships and to stimulate further work on nonlinear behavior, complex formulations, and validated, uncertainty-aware process models.
